# Effects of Immunomodulatory Substances on Phagocytosis of A*β*_1–42_ by Human Microglia

**DOI:** 10.4061/2010/798424

**Published:** 2010-05-20

**Authors:** Erik Hjorth, Dan Frenkel, Howard Weiner, Marianne Schultzberg

**Affiliations:** ^1^Division of Neurodegeneration, Department of Neurobiology, Care Sciences and Society, Karolinska Institutet, 141 86 Stockholm, Sweden; ^2^Department of Neurobiology, Tel Aviv University, Tel Aviv 69978, Israel; ^3^Center for Neurologic Disease, Brigham & Womens Hospital, Harvard Medical School, 75 Francis Street, Boston, MA 02115, USA

## Abstract

Glial activation and increased inflammation characterize neuropathology in Alzheimer's disease (AD). The aim was to develop a model for studying phagocytosis of *β*-amyloid (A*β*) peptide by human microglia and to test effects thereupon by immunomodulatory substances. Human CHME3 microglia showed intracellular A*β*_1–42_ colocalized with lysosome-associated membrane protein-2, indicating phagocytosis. This was increased by interferon-*γ*, and to a lesser degree with Protollin, a proteosome-based adjuvant. Secretion of brain-derived neurotrophic factor (BDNF) was decreased by A*β*_1–42_ and by interferon-*γ* and interleukin-1*β*. These cytokines, but not A*β*_1–42_, stimulated interleukin-6 release. Microglia which phagocytosed A*β*_1–42_ exhibited a higher degree of expression of interleukin-1 receptor type I and inducible nitric oxide synthase. In conclusion, we show that human microglia are able to phagocytose A*β*_1–42_ and that this is associated with expression of inflammatory markers. A*β*_1–42_ and interferon-*γ* decreased BDNF secretion suggesting a new neuropathological role for A*β*_1–42_ and the inflammation accompanying AD.

## 1. Introduction

Alzheimer's disease (AD) is the most common cause of dementia. The major pathological hallmarks of AD besides neuronal loss are amyloid plaques and neurofibrillary tangles (NFTs). Both amyloid plaques and NFTs have been implicated in neuronal impairment and death in a large number of studies. Although a great deal of controversy exists about the relative importance of amyloid plaques *contra* NFTs in AD [1], there is an overwhelming body of evidence showing that amyloid plaques and the peptides they are composed of are culprits in the neurodegenerative processes in AD [2]. The main component of amyloid plaques is the *β*-amyloid (A*β*) peptide, that is secreted by neurons and other cells through cleavage of the larger, membrane-bound, amyloid precursor protein (APP). APP can be processed by two major pathways: the amyloidogenic pathway that yields A*β*, and the nonamyloidogenic yielding fragments believed to be nonpathogenic. The A*β* peptides are prone to self-aggregation and deposition into insoluble plaques. This is especially true for the 42 amino acid form (A*β*_1*–*42_), which is the predominant form in the dense core plaques [3]. A*β*-species also exist in the forms of soluble monomers and oligomers which similarly to the amyloid plaques are more abundant in the AD brain than in the non-AD brain [4]. Somehow, the balance between production and clearance/degradation of A*β* is disturbed in AD. 

Damage to brain tissue induces an inflammation, a response which is present in many, if not all neurodegenerative conditions. In the central nervous system (CNS), glial cells, that is, microglia and astrocytes, represent the main source of inflammatory reactions. In normal conditions, glia have supportive functions, including maintenance of ionic homeostasis, clearance of neurotransmitters, and in the case of astrocytes, providing nutrients to the energy-demanding work of the neurons [5]. Glial, particularly microglial, cell responses can also serve in the elimination of debris from damaged cells and to remove pathogens. Removal of pathogens is executed by the process of phagocytosis, a capability that glial cells share with peripheral immunocompetent cells including monocytes and macrophages. In the brain, phagocytosis is believed to be performed mainly by microglia, but astrocytes also have this capability [6].

Under stress, glial cells proliferate and become activated, which leads to production of neurotoxic molecules such as free radical species and proinflammatory cytokines [7, 8]. In contrast to the potentially tissue-damaging responses, glia can also produce factors that promote neuroprotection and neuronal plasticity [9]. These protective and supportive functions may be downregulated during stress. Therefore, inflammation in the brain may worsen the outcome of an already existing trauma or pathological condition.

In the AD brain, activated microglia and astrocytes are present in the areas of neurodegeneration and amyloid plaques [10] and there is an increased production of proinflammatory cytokines such as interleukin- (IL)-1 [11] and IL-6 [12]. In addition, elevated levels of IL-1 and IL-6 have been found in serum and cerebrospinal fluid (CSF) from AD patients [13, 14]. In vitro studies have demonstrated that A*β* peptides can indeed activate glial cells to produce inflammatory factors [15–18], which can contribute to the neurodegenerative process [19]. Evidence from studies on rat cortical microglia showed that the smaller aggregational forms of A*β* such as oligomers are more potent in stimulating glial secretion of proinflammatory cytokines [18]. IL-1 increases the production of APP in human glia [20], and the APP gene contains a binding site for the prime inflammatory transcription factor nuclear factor *κ*B (NF*κ*B) [21]. Furthermore, inflammation may shift processing of APP towards the amyloidogenic pathway [22]. A consequence of the interactions between inflammation and the APP/A*β*-peptide may be a vicious circle [23], in which inflammation increases A*β* levels through increased production and reduced clearance, which in turn results in neuronal cell death and a perpetuated and increased glial activation and release of proinflammatory factors, as well as neuronal cell death, and so forth.

Removal of a disease-causing pathogen is probably the most effective way of treating a disease. To stimulate cellular uptake, phagocytosis of A*β* is a promising strategy. In studies on animal models of AD, active and passive immunizations have been shown to be effective in removing plaques and to improve cognitive performance [24]. Human clinical trials with active immunization have been started but were aborted due to serious side-effects in a few cases [25]. However, several clinical trials with modified protocols, including passive immunizations, are currently being carried out. Phagocytosis is an activity that is performed primarily by cells of the immune system, notably cells of the monocyte lineage. In the light of the studies on active and passive immunization we are presented with a strategy for treatment of AD, to activate the immune system into phagocytosis of A*β*. Although inflammation generally is believed to stimulate phagocytic activities, there is also evidence indicating that inflammation may inhibit phagocytosis [26], whereas anti-inflammatory cytokines such as transforming growth factor-*β* (TGF-*β*) can stimulate phagocytosis [27]. Thus, it is of importance to search for compounds that can promote phagocytosis without starting the damaging processes of inflammation. In short, directed and differential activation of immune cells residing in, or destined for, the CNS constitutes a potential therapeutic target.

In the present study, the aim was to characterise the responses of human microglia to the exposure of A*β*. In order to investigate the possibilities to increase glial uptake of A*β* we have analysed the effects of different immunomodulatory substances. The effects of the adjuvant Protollin, as well as those of the archetypical proinflammatory cytokines, IL-1*β* and interferon-*γ* (IFN*γ*), were analysed in an in vitro model of human microglial cells with regard to A*β*_1*–*42_ uptake, microglial phenotype, and the secretion of IL-6 and brain-derived neurotrophic factor (BDNF). 

IL-6 is a cytokine that is induced by IL-1*β* and tumour necrosis factor-*α* (TNF*α*) and can thus be considered a general measure of inflammation [28]. BDNF is a neurotrophic growth factor of importance in memory formation and neuroprotection [29, 30]. Protollin is an adjuvant made of *Shigella flexneri* 2a lipopolysaccharides (LPS) associated noncovalently to meningococcal outer membrane proteins (proteosomes), that has been proven to be safe for use in humans [31]. While LPS activates Toll-like receptor type 4 (TLR4), cd11, and cd14, proteosomes activate TLR2 [32]. Activation of TLR2 has been associated with increased phagocytosis in mice with sciatic nerve lesions [33]. Furthermore, intranasal application of Protollin was shown to prevent accumulation of A*β* in young transgenic mice expressing the human APP with the Swedish mutation and also to stimulate clearance of A*β* from the brain of aged mice of the same transgenic strain [34]. A correlation was found between the removal of A*β* and the level of microglial activation, as demonstrated by increased expression of the activation marker CD11b in conjunction with the removal of A*β* in animals treated with Protollin [34]. To investigate the effects of Protollin on cellular inflammatory markers, and the association of these markers with uptake of A*β*_1*–*42_ by the human microglial cells, the expression of inducible nitric oxide synthase (iNOS), IL-1*β*, and the signalling type I receptor for IL-1*β* (IL-1RI) was analyzed in cells showing uptake of A*β*_1*–*42_. iNOS is induced in inflammation and shown to be harmful for neurons due to the production of radical nitrogen species [35]. It has been shown to be associated with neurodegenerative disorders such as AD [36].

## 2. Materials and Methods

### 2.1. Chemicals

Protollin was provided by Glaxo-Smith Kline Biologicals, Laval, Quebec, Canada. A*β*_1*–*42_ conjugated with HiLyteFluor488 or biotin was obtained from Anaspec (Fremont, USA). Dimethylsulfoxide (DMSO), Triton-X100, bovine serum albumin (BSA), 4′,6-diamidino-2-phenylindole (DAPI), and (3-(4,5-dimethylthiazol-2-yl)-2,5-diphenyltetrazolium bromide (MTT) were purchased from Sigma, Stockholm, Sweden. Normal donkey and goat serum and fluorescence mounting medium were from DakoPatts (Stockholm, Sweden). Streptavidin-7-amino-4-methyl-3-coumarinylacetic acid (AMCA) is from Jackson ImmunoResearch Europe Ltd (Suffolk, UK). Lactate deydrogenase (LDH) assay is from Roche (Stockholm, Sweden). ELISA-kits for IL-6 and BDNF are from R&D systems (Abingdon, United Kingdom). IFN*γ* (Bachem, Weil am Rhein, Germany). Cell culture medium, phosphate-buffered saline (PBS), GlutaMaxII, foetal calf serum (FCS) and PBS-based enzyme-free cell dissociation buffer (Invitrogen, Stockholm, Sweden). Cell culture bottles and multiwell plates (BD Biosciences, Stockholm, Sweden).

### 2.2. Cell Cultures

Human microglial cells (CHME3) were obtained as a kind gift from Professor M. Tardieu (Neurologie pédiatrique, Hôpital Bicêtre, Assistance publique, Hôpitaux de Paris, Paris, France). CHME3 cells were cultured in T75 or T175 bottles in culture medium (DMEM/high glucose w/o sodium pyruvate supplemented with 2 mM L-glutamine or GlutaMaxII and 10% heat-inactivated FCS). The cells were subcultured at confluence using enzyme-free cell dissociation buffer after washing once with PBS without Mg^2+^ and Ca^2+^.

### 2.3. Experimental Procedures

The CHME3 microglial cells were seeded in 48-well plates for analysis of cell viability (MTT) and cytotoxicity (LDH), in 6-well plates for flow-cytometry and analysis of substances released to the medium, or on glass coverslips in 24- or 48-well plates for immunocytochemistry. All experiments were performed at a confluence of ~60%. A*β*_1*–*42_ was dissolved in DMSO and stored in darkness at +4°C until use at a final concentration of 1 *μ*g/mL for all experiments except for a dose-response curve. In higher doses we observed aggregate-like precipitates of fluorescent A*β*_1*–*42_ which were unwanted in this study. Before the addition of A*β*_1*–*42_ or vehicle (DMSO), the cells were prestimulated for 24 hours with either Protollin (0.001, 0.01 and 0.1 *μ*g/mL in serum-free culture medium) or cytokines (50 ng/mL IL-1*β*, 50 ng/mL IFN*γ*, or IL-1*β* + IFN*γ*). At 0, 24, 48, 72, and 96 hours after addition of A*β*_1*–*42_ the cultures were analyzed for uptake of A*β*_1*–*42_, expression of cellular markers, and secretory products. Uptake of fluorescent A*β*_1*–*42_ by living cells was analysed in a Nikon TE600-inverted fluorescence microscope. Cell viability and cell death were also analysed.

### 2.4. Quantification of A*β*_1–42_ Phagocytosis and Cellular Markers by Flow-Cytometry

After the experimental treatment, the CHME3 microglial cells were dissociated with PBS-based enzyme-free dissociation buffer as described above, and centrifuged at 1500 ×g for 10 minutes. The cells were then resuspended and fixed in 1% para-formaldehyde (PF) in PBS, for 40 minutes at room temperature, after which the fixative was diluted 20× by addition of PBS and the cells were centrifuged at 1500 ×g for 10 minutes. Fixation with PF renders cells permeable to PI and therefore all fixed cells will be stained with PI, allowing the distinction of cells from cell debris. Analysis was performed in a FACScalibur (BD) flow-cytometer. A detected event was defined as a cell if it was gated through the front-scatter (FSC) and side-scatter (SSC) plot gate in addition to being positive for PI. A cell positive for phagocytosis of A*β*_1*–*42_ (A*β*_1*–*42_+) was defined as a PI-positive cell that was also being positive for the fluorophore conjugated to A*β*_1*–*42_ (HyliteFluor488). Negative controls were utilized to establish the limits of detection for positive signals. 

To investigate the phenotype of the cells with regard to the presence of inflammatory markers, and the degree of colocalization of each marker with phagocytosed A*β*_1*–*42_, the cells were stained with antibodies directed against human IL-1*β* (1 : 400; gift from Dr. Stefan Svensson, Statens Bakteriologiska Laboratorium, Stockholm, Sweden), IL-1RI (1 : 200; Amgen (Immunex Corporation) Thousand Oaks, USA), and iNOS (1 : 600; R&D systems, London, England). The cells were harvested and fixed as described above and subsequently an aliquot of the cell suspension was incubated with the primary antibodies, diluted in PBS containing 5% normal donkey serum and 0.1% Triton-X100. Omission of primary antibodies served as negative control to establish the limits of detection for positive signals. After incubation with primary antibodies overnight at +4°C, the cell suspension was washed by addition of PBS followed by centrifugation at 2500 ×g for 20 minutes. The cells were resuspended in PBS and incubated with donkey antigoat IgG-NL637 antibodies (1 : 500; R&D systems, London, England) and PI for 1 hour at room temperature. After incubation, the cell suspension was diluted with PBS and analyzed by flow-cytometry. A cell displaying immunoreactivity for an antibody was defined as a cell showing a stronger signal in this channel than the negative control. A cell displaying uptake of A*β*_1*–*42 _ (A*β*_1*–*42_+) was defined as described above. The results were analysed from a scatter plot with fluorescence from the secondary antibody on the *y*-axis and fluorescence from HyliteFluor488 on the *x*-axis, divided into quadrants with borders based on the negative controls as described. The cells were thus being viewed in a two-way binary fashion with a cell being present in a certain quadrant thus indicating (a) positive for immunoreactivity to IL-1*β* *(*IL-1*β*+), IL-1RI (IL-1RI+), or iNOS (iNOS+) and for A*β*_1*–*42_-uptake (A*β*_1*–*42_+), (b) positive for immunoreactivity to one of these markers and negative for A*β*_1*–*42_-uptake, (c) negative for immunoreactivity to the markers and positive for A*β*_1*–*42_-uptake, or (d) negative for immunoreactivity to the markers and for A*β*_1*–*42_-uptake. The parameters extracted from the data were (a) total proportion of the cells showing immunoreactivity to a marker, (b) immunoreactivity of cells negative for A*β*_1*–*42_-uptake, and (c) immunoreactivity of cells positive for A*β*_1*–*42_-uptake.

### 2.5. Immunocytochemistry and Staining of Fixed and Living Cells for Microscopy

To analyse the microglial cell expression of certain inflammatory markers and the localization of phagocytosed A*β*_1*–*42_ by microscopy the culture medium was removed and the coverslips dried at 37°C for ~2 hours. The cells were fixed with 4% PF for 20 minutes at room temperature, washed in PBS, and incubated overnight at 4°C with antibodies against IL-1*β* (1 : 400), IL-1RI (1 : 200), and iNOS (1 : 600), respectively. The antibodies were diluted in PBS containing 5% normal donkey serum and 0.1% Triton-X100. After rinsing in PBS, the coverslips were incubated for 1 hour at room temperature with goat antirabbit-IgG conjugated with Cy2 (1 : 200), diluted in PBS containing 5% normal goat serum, 0.1% Triton-X100, and 1 *μ*g/mL PI. For visualization of biotinylated A*β*_1*–*42_, streptavidin-AMCA was included in the secondary antibody solution (2 *μ*g/mL). The coverslips were then washed with PBS, mounted with fluorescence mounting medium, and inspected in a Nikon E800 microscope. To investigate the targeting of A*β*_1*–*42_ toward degradation, cells were incubated with HyliteFluor488-conjugated A*β*_1*–*42_ and then fixed and incubated overnight at 4°C with mouse antibodies against human lysosome-associated membrane protein-2 (lamp-2, Millipore). After washing, the cells were incubated with donkey antimouse antibodies conjugated with Cy3 and with the nuclear stain DAPI and then mounted and inspected as described above.

### 2.6. Enzyme-Linked Immunosorbent Assay (ELISA)

The levels of IL-6 and BDNF in the cell culture medium were analyzed with commercially available ELISA-kits according to the manufacturer's instructions. Analysis of optical density (OD) was performed in a TECAN Safire2 plate reader.

### 2.7. Cell Viability—MTT Assay

To analyse cell viability the culture medium was removed and after washing with serum- and phenol-free DMEM/high glucose with GlutaMaxII, MTT (0.3 mg/mL) was added and the cells incubated at 37°C for 1 hour. The crystals formed by the reaction were dissolved in DMSO and the OD was measured with a TECAN Safire2 plate reader (Tecan, Mölndal, Stockholm) at 592 nm with 620 nm as a reference wavelength.

### 2.8. Cell Death—LDH Assay

To analyse cell death the culture medium was removed and added to a 96-well plate and then incubated with LDH-reagent according to the manufacturer's instructions. The OD was measured at 492 nm with 620 nm as reference wavelength.

### 2.9. Statistics

The data were normalized to vehicle (MTT-assay, ELISA) A*β*_1*–*42_ (flow-cytometry, for uptake) or positive control (LDH-assay), or not normalized (flow-cytometry, for association of cellular markers with the uptake of A*β*_1*–*42_, correlation between variables). The results were analysed with Kruskal-Wallis nonparametric analysis of variance (ANOVA) and the data were then compared pairwise using the nonparametric Mann-Whitney U test with Bonferoni correction. Pairwise comparison of variable within experimental groups was performed with the Wilcoxon Matched Pairs Test. Correlations between variables were analysed with the Spearman's Rank Order Correlation test. All statistical analyses were performed in Statistica v8 (Statsoft).

## 3. Results

Human CHME3 microglial cells exposed to immunomodulatory substances were studied with regard to phagocytosis of A*β*_1*–*42_ and expression of cellular markers (IL-1*β*, IL-1RI, and iNOS). As an indicator of an inflammatory response we analysed the secretion of IL-6 in the culture medium. To assess beneficial and neuroprotective activities the secretion of BDNF was determined. The levels of BDNF released into the medium varied between 8 and 300 pg/mL and the levels of IL-6 varied between 20 and 950 pg/mL under basal conditions (vehicle group at 24 h). Cell death and viability were also analysed. 

**Figure 1:**
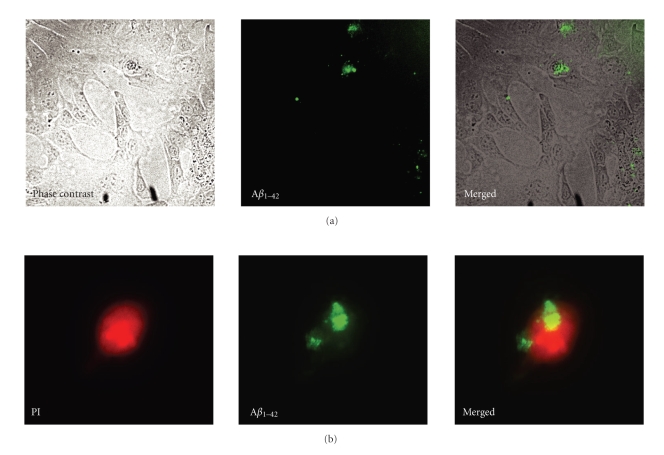
(a)-(b) Uptake of A*β*_1*–*42_ in human microglial cells. The micrographs in (a) show living human CHME3 microglial cells in culture after incubation with A*β*_1*–*42_, seen by phase contrast and fluorescence microscopy, and after merging of these. The micrographs in (b) show fixed CHME3 microglial cells in suspension after incubation with A*β*_1*–*42_, seen by fluorescence microscopy with filters for propidium iodide (PI) staining and HiLyte488-conjugated A*β*_1*–*42_, respectively, and the micrographs are merged in the 3rd micrograph. Magnifications 10× (a) and 40× (b).

**Figure 2:**
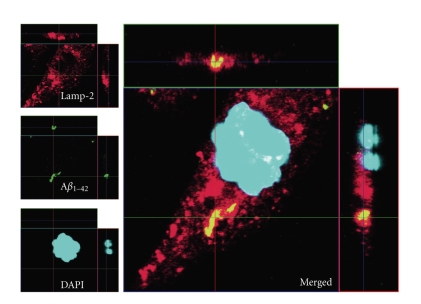
Uptake of A*β*_1*–*42_ in human microglial cells. The confocal micrograph (63×) shows fixed CHME3 microglial cells demonstrating intracellular lysosomal location of HiLyte488-conjugated A*β*_1*–*42_ (green filter). Lysosomes were visualized by staining with an antibody against lysosome-associated membrane protein-2 (lamp-2) and Cy3-conjugated secondary antibodies (red filter). Yellow colour thus indicates colocalization of lamp-2 and HiLyte488-conjugated A*β*_1*–*42_.

**Figure 3:**
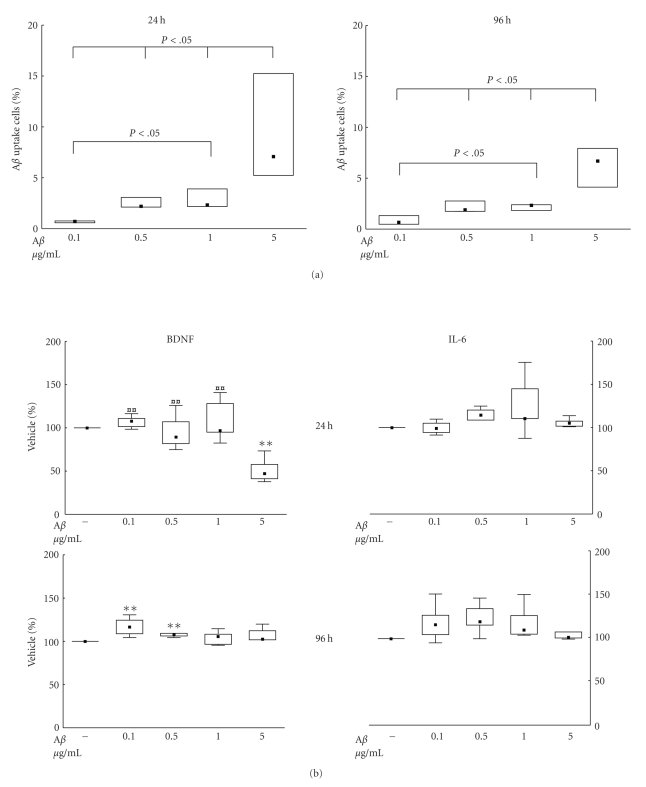
(a)-(b) Uptake of A*β*_1*–*42_ (a) and the concurrent secretion of interleukin-6 (IL-6) and brain-derived neurotrophic factor (BDNF) (b) by human CHME3 microglial cells at 24 and 96 hours. The cells were incubated with concentrations of A*β*_1*–*42_, ranging between 0.1 and 5 *μ*g/mL, or vehicle. The cells were harvested and the medium collected at 24 and 96 hours. In (a), the data are expressed as the proportion of cells with intracellular A*β*_1*–*42_, *n* = 4. In (b), the data are expressed as % of control (vehicle) set at 100% and shown as median ± percentiles (25%–75% and 10%–90%). There was a significant effect of the treatment on the uptake of A*β*_1*–*42_ at 24 hours (*P* = .0045) and at 96 hours (*P* = .0375). The secretion of BDNF was also significantly altered by the treatment at 24 hours (*P* = .0102) and at 96 hours (*P* = .0375). Statistical difference from A*β*_1*–*42_, 5 *μ*g/mL, is indicated by 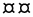
(*P* < .01); statistical difference from vehicle is indicated by **(*P* < .01).

The ability of CHME3 microglia to take up A*β*_1*–*42_ was confirmed by inspection of living cells using phase contrast/fluorescence microscopy (Figure 1(a)), and fluorescence microscopy on the fixed cell suspension used for flow-cytometry (Figure 1(b)), and by confocal microscopy on fixed and living cells (Figure 2). The first signs of uptake (visible in inverted fluorescence microscope) by the microglial cells were observed approximately 4 hours after the addition of A*β*_1*–*42_. Analysis of the intracellular content of A*β*_1*–*42_ was performed by flow-cytometry at 24 and 96 hours after addition of different concentrations of A*β*_1*–*42_ (Figure 3(a)). The uptake increased with increasing concentrations of A*β*_1*–*42_. A 50-fold increase in the concentration of A*β*_1*–*42_ from 0.1 to 5 *μ*g/mL resulted in a 10-fold increase in the fraction of cells taking up A*β*_1*–*42_ (*P* < .05).

### 3.1. Effects of A*β*_1–42_ on Secretion of BDNF and IL-6

In dose-response experiments with 0.1, 0.5, 1, and 5 *μ*g/mL of A*β*_1*–*42_, we found that 5 *μ*g/mL reduced the levels of BDNF in the culture medium by 50% compared to vehicle (*P* < .01, Figure 3(b)). A similar degree of decrease was seen for 5 *μ*g/mL A*β*_1-42_ when compared with the other concentrations of A*β*_1*–*42_ (*P* < .01). At 96 hours, the concentrations of 0.1 and 0.5 *μ*g/mL A*β*_1*–*42_ slightly increased the secretion of BDNF (*P* < .05). No effect was seen on the secretion of IL-6 with the concentrations of A*β*_1*–*42_ tested (Figure 3(b)).

### 3.2. Effects of IL-1*β* and IFN*γ* on Uptake of A*β*_1–42_

**Figure 4:**
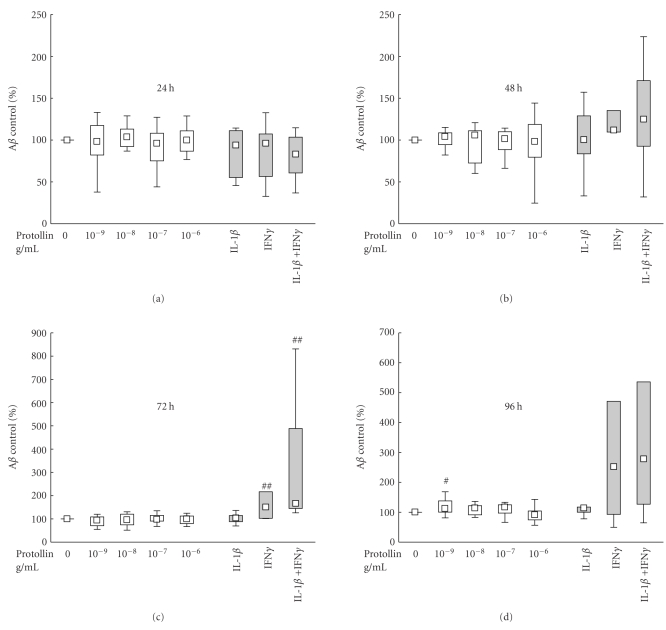
Effects of Protollin (white boxes) and interleukin-1*β* (IL-1*β*) and interferon-*γ* (IFN*γ*) (grey boxes) on the uptake of A*β*_1*–*42_ in human CHME3 microglial cells. The cells were incubated with 1 *μ*g/mL A*β*_1*–*42_ following prestimulation for 24 hours with Protollin at 0.001–1 *μ*g/mL, or 50 ng/mL IL-1*β* and 50 ng/mL IFN*γ*. The cells were harvested at 24, 48, 72, and 96 hours after addition of A*β*_1*–*42_. The data are expressed as % uptake of control A*β*_1*–*42_ set at 100%, and shown as median ± percentiles (25%–75% and 10%–90%), *n* = 19 (Protollin) or *n* = 4 (cytokines) for 24 and 48 hours, and *n* = 17 (Protollin) of *n* = 6 (cytokines) for 72 and 96 hours. A statistically significant effect of treatment was found by Kruskal-Wallis ANOVA at 96 hours when incubating microglia with Protollin (*P* = .0157) and at 72 hours when incubating with IL-1*β* and/or IFN*γ* (*P* = .0022). Statistical difference from control is indicated by ^#^(*P* < .05), ^##^(*P* < .01), and ^###^(*P* < .005) indicates statistical difference (*P* < .01) between IL-1*β* and IL-1*β* + IFN*γ*.

To evaluate the potential of stimulating CHME3 microglia into phagocytosis by inflammation, the cells were incubated with the proinflammatory cytokines IL-1*β* and IFN*γ*. Pretreatment with IFN*γ* resulted in an increase in the proportion of cells showing A*β*_1*–*42_ uptake (A*β*_1*–*42_+ cells) with a median increase of 50% at 72 hours (*P* < .01, Figure 4, grey boxes), and the combined stimulation with IL-1*β* and IFN*γ* lead to a median increase of 66% at 72 hours (*P* < .01) as compared to controls (with A*β*_1*–*42_ alone) and a 62% increase compared to IL-1*β* (*P* = .05). IL-1*β* alone had no significant effect on the A*β*_1*–*42_ uptake. The observed effects appeared to linger at 96 hours although the differences were not significant.

### 3.3. Effects of IL-1*β* and IFN*γ* on Secretion of BDNF and IL-6

**Figure 5:**
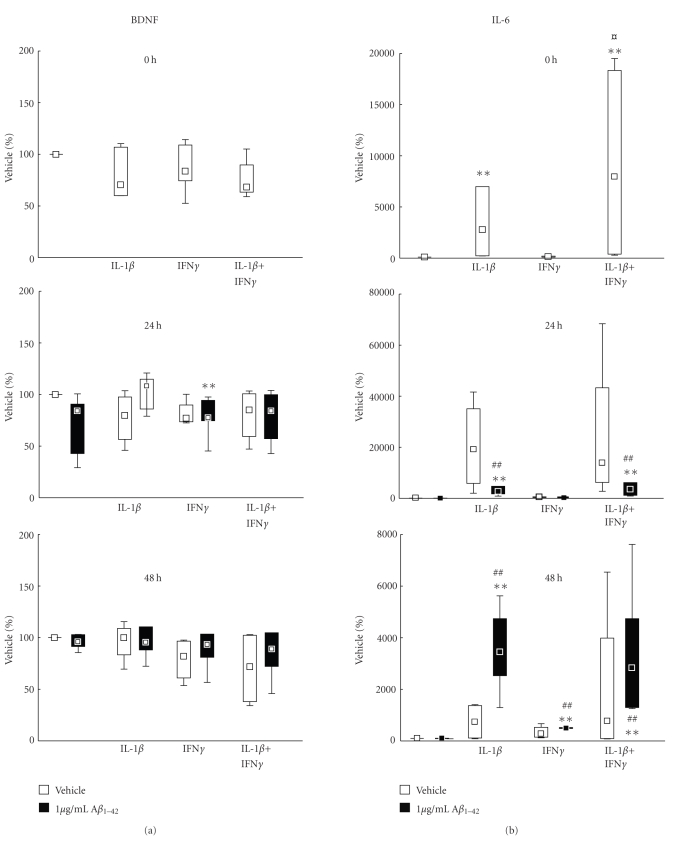

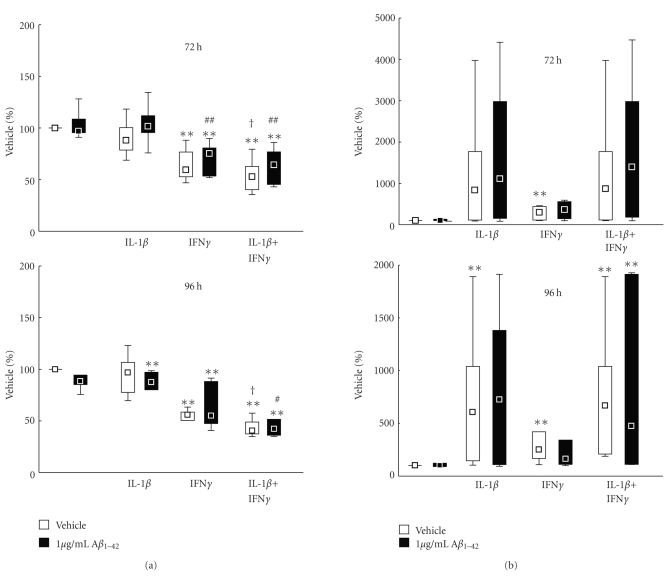
(a)-(b) Effects of incubation with interleukin-1*β* (IL-1*β*) and interferon-*γ* (IFN*γ*), added 24 hours before incubation with 1 *μ*g/mL A*β*_1*–*42_ (black boxes) or vehicle (white boxes), on secreted levels of IL-6 (a) and BDNF (b) from CHME3 microglial cells. The cells were incubated with 1 *μ*g/mL HiLyte488-conjugated A*β*_1*–*42_ following prestimulation for 24 hours with 50 ng/mL IL-1*β* and 50 ng/mL IFN*γ*. The cells were harvested at 24, 48, 72, and 96 hours after addition of A*β*_1*–*42_. The data are expressed as % uptake of control vehicle set at 100% and shown as median ± percentiles (25%–75% and 10%–90%), *n* = 4 for 24 hours and 48 hours and *n* = 6 for 72 and 96 hours. A statistically significant effect of treatment was found by Kruskal-Wallis ANOVA at 24 hours (*P* = .0087), at 72 hours (*P* = .004), and at 96 hours (*P* = .004). Statistical difference from control is indicated by **(*P* < .01); difference from A*β*_1*–*42_ # is indicated by (*P* < .05) and ^##^(*P* < .01). † indicates statistical difference (*P* < .05) between IL-1*β* and IL-1*β* + IFN*γ*.

The levels of secreted BDNF were markedly reduced by the incubation with the proinflammatory cytokines IL-1*β* and IFN*γ* (Figure 5(a), white boxes). Thus, IFN*γ* decreased the median secretion of BDNF as compared to vehicle with 41% at 72 hours and with 44% at 96 hours (*P* < .05 at both time points). There was no effect of IL-1*β* alone, but the combination of IL-1*β* and IFN*γ* resulted in a significant, almost 50%, decrease in BDNF secretion at 72 and 96 hours (*P* < .05 at both time points) (Figure 5(a), white boxes).

Treatment of the cells with proinflammatory cytokines resulted in a marked inflammatory response evidenced by a dramatic increase in IL-6 secretion (Figure 5(b), white boxes). At 0 hour (i.e., 24 hours after addition of the cytokines and at the time point of addition of A*β*_1*–*42_, or in this case, vehicle), IL-1*β* and IL-1*β* + IFN*γ* stimulated the IL-6 secretion, producing levels that were 50 and 90 times higher than control (vehicle), respectively (*P* < .05 in both cases (Figure 5(b), white boxes). At 24 hours (i.e., after 48 hours incubation with the cytokines), IL-1*β* and IL-1*β* + IFN*γ* induced a 200- and 140-fold increase in IL-6 secretion, respectively, and the combination of IFN*γ* and IL-1*β* resulted in a 25× higher induction than that by IFN*γ* alone (*P* < .05). The incubation with IL-1*β* + IFN*γ* was still effective at 96 hours in increasing secreted levels of IL-6 (*P* = .0108). At this time point, IL-1*β* alone and IFN*γ* alone significantly increased the levels of IL-6 several-fold (*P* < .05 in both cases).

### 3.4. Effects of IL-1*β* and IFN*γ* in Combination with A*β*_1–42_ on Secretion of BDNF and IL-6

Similarly to the effect seen for cytokines, the combined incubation of cytokines and A*β*_1*–*42_ resulted in reduced secretion of BDNF (Figure 5(a), black boxes). There was no effect of A*β*_1*–*42_ alone on secretion of BDNF in this series of experiments. At 24 hours, pretreatment with IFN*γ* resulted in a median reduction to 77% in BDNF secretion compared to vehicle (100%) (*P* = .0086). At 72 hours, the median reduction was 25% (*P* < .01) and at 96 hours it was 45% (*P* < .01) (Figure 5(a), black boxes). The combination of A*β*_1*–*42_ with IFN*γ* pretreatment resulted in significantly lower secretion of BDNF than that observed after treatment with A*β*_1*–*42_ alone at 72 hours (*P* < .01). Pretreatment with IL-1*β* decreased the median BDNF secretion to 88% of vehicle at 96 hours (*P* < .01). At 72 hours, pretreatment with IL-1*β* + IFN*γ* resulted in a significant decrease in BDNF secretion to 64% of vehicle secretion (*P* < .01) which was also lower than the secretion induced by A*β*_1*–*42_ alone at this time point (97% of vehicle, *P* < .01). This effect was still present at 96 hours when pretreatment with IL-1*β* + IFN*γ* resulted in a decrease in BDNF level to 43% of vehicle (*P* < .01), which also at this time point was lower than the secretion induced by A*β*_1*–*42_ alone (88% of vehicle, *P* = .017). The pretreatment with IL-1*β* + IFN*γ* also reduced the secretion of BDNF compared with IL-1*β* at 72 hours (*P* < .05).

Treatment of the cells with cytokines before addition of A*β*_1*–*42_ (Figure 5(b), black boxes) resulted in a marked increase in IL-6 secretion that paralleled the increase observed in the absence of A*β*_1*–*42_ (Figure 5(b), white boxes). In this series of experiments there were no effects of A*β*_1*–*42_ alone at any time point on the secretion of IL-6. The pretreatment of the CHME3 microglial cells with IL-1*β* or IL-1*β* + IFN*γ* had a strong stimulatory effect on the secretion of IL-6 as compared to vehicle and to A*β*_1*–*42_ alone (Figure 5(b), black boxes). At 24 hours, IL-1*β* increased the median IL-6 secretion 25-fold compared with vehicle and A*β*_1*–*42_ alone (*P* < .01 in both cases). A significant stimulatory effect of IL-1*β* pretreatment was seen at 48 hours with levels 35-fold increase as compared to vehicle and A*β*_1*–*42_ alone (*P* < .01 in both cases). At 48 hours, a stimulatory effect of IFN*γ* became apparent, with a 5-fold increase in IL-6 secretion compared with vehicle and A*β*_1*–*42_ alone (*P* < .01 in both cases). Compared with vehicle and A*β*_1*–*42_ alone, the combined pretreatment with IL-1*β* + IFN*γ* induced a 35-fold increase at 24 hours (*P* < .01 in both cases) and a 28-fold increase at 48 hours (*P* < .01 in both cases). A 5-fold increase was seen with the combined IL-1*β* + IFN*γ* treatment at 96 hours compared with vehicle (*P* < .01). Also, the combined stimulation with IFN*γ* and IL-1*β* prior to A*β*_1*–*42_ resulted in an increase of almost 6 times in the mean secretion of IL-6 at 48 hours as compared to IFN*γ* alone (*P* < .005), but not compared to IL-1*β*.

### 3.5. Effects of Protollin on Uptake of A*β*_1–42_

Pretreatment of the microglial cells with Protollin at 0.001 *μ*g/mL was found to increase the median proportion of cells showing uptake of A*β*_1*–*42_ to 115% (*P* < .05) as compared to control (A*β*_1*–*42_ alone, 100%, Figure 4). This effect was seen at 96 hours, but no significant changes in any direction could be detected at the earlier time points.

### 3.6. Effects of Protollin, with and without A*β*_1–42_, on Secretion of BDNF and IL-6

Treatment with Protollin alone, that is, prior to addition of vehicle, did not affect the secretion of BDNF (data not shown). The incubation with A*β*_1*–*42_ alone resulted in a decrease in the secretion of BDNF in this series of experiments, that is, a reduction to 82% of vehicle at 24 hours (*P* < .0000001), to 77% of vehicle at 48 hours (*P* < .005) and to 95% of vehicle at 96 hours (*P* < .05).

In cultures pretreated with the lowest concentration of Protollin (0.001 *μ*g/mL) followed by incubation with A*β*_1*–*42_ there was a 22% reduction at the 24 hours time point as compared to vehicle (*P* = .05), whereas at the higher concentrations of Protollin and at later time points there was no significant differences in the secretion of BDNF in comparison with control conditions (no Protollin and no A*β*_1*–*42_).

Pretreatment with Protollin followed by A*β*_1*–*42_ or vehicle did not induce any significant effects on the levels of IL-6 in culture supernatants (data not shown).

### 3.7. Cellular Markers and Relation to Phagocytosis of A*β*_1–42_

**Figure 6:**
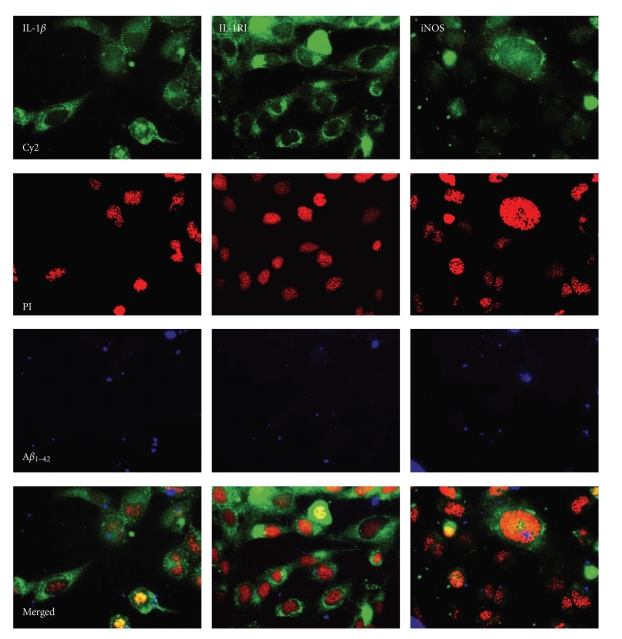
Uptake of A*β*_1*–*42_ and expression of cellular markers in human microglial cells. The micrographs show human CHME3 microglial cells after incubation with biotinylated A*β*_1*–*42_, after which they were fixed and stained with antibodies against interleukin-1*β* (IL-1*β*), IL-1 receptor type I (IL-1RI) and inducible nitric oxide synthase (iNOS), and subsequent incubation with Cy2-conjugated secondary antibodies. Cell nuclei were stained with propidium iodide (PI) and the biotinylated A*β*_1*–*42_ was visualized with AMCA-conjugated streptavidin. All micrographs are in 20× magnification.

**Figure 7:**
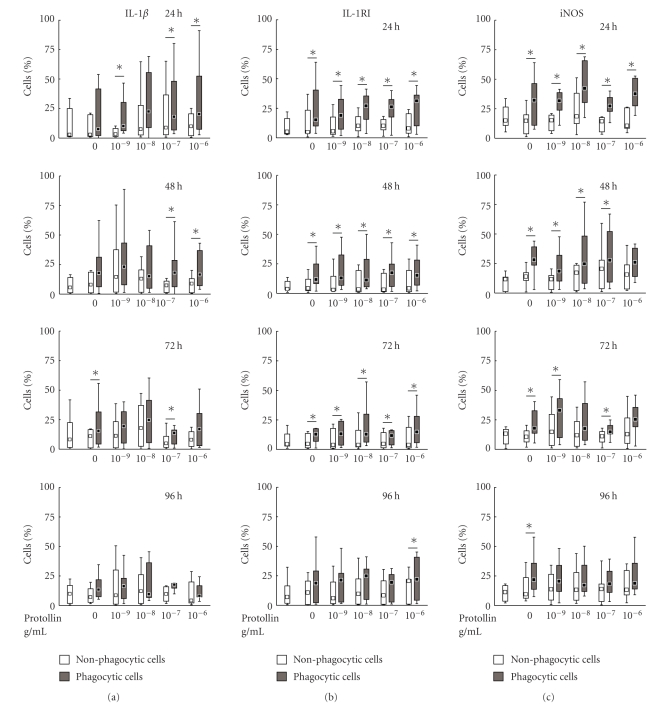
(a)–(c) Differential phenotype of human CHME3 microglia, in the populations of cells displaying (A*β*_1*–*42_+, grey boxes) or not displaying (A*β*_1*–*42_−, white boxes) phagocytosis of A*β*_1*–*42_, with regard to immunoreactivity for interleukin-1*β* (IL-1*β*), inducible nitric oxide synthase (iNOS), and IL-1*β* receptor type I (IL-1RI), following pretreatment with Protollin (0.001–1 *μ*g/mL). After 24–96 hours of exposure to A*β*_1*–*42_, the cells were subjected to immunocytochemistry and analysed by flow-cytometry. The population of cells with immunoreactivity to the different markers within the A*β*_1*–*42_+ cell population was compared with the corresponding population in the A*β*_1*–*42_− cell population in each treatment group, using the Wilcoxon Matched Pairs Test. The data are shown as median ± percentiles (25%–75% and 10%–90%), *n* = 7. Statistical differences between the A*β*_1*–*42_+ and A*β*_1*–*42_− cells with regard to each marker are indicated by *(*P* < .05).

The uptake of A*β*_1*–*42_ in cells expressing the inflammatory markers IL-1*β*, IL-1RI, and iNOS in the cultures treated with A*β*_1*–*42_ following pretreatment with Protollin was demonstrated by immunocytochemistry (Figure 6). The proportion of cells positive for these markers was analysed by flow-cytometry, both after incubation with A*β*_1*–*42_ alone and after pretreatment with Protollin, in order to assess the phenotype of the cells taking up A*β*_1*–*42_ (A*β*_1*–*42_+) (Figure 7).

### 3.8. IL-1*β* Immunoreactive Cells

In untreated (vehicle) cultures, the proportion of CHME3 microglial cells with immunoreactivity to IL-1*β* had a median of 2.9% at 24 hours. Incubation of the cells with 1 *μ*g/mL A*β*_1*–*42_ did not significantly affect the number of IL-1*β* immunoreactive cells compared with vehicle at any time point (Figure 7(a)).

The expression of IL-1*β* in A*β*_1*–*42_+ cells was not significantly different from that in A*β*_1*–*42_− cells according to the Wilcoxon Matched Pairs test, except at 72 hours when the population of A*β*_1*–*42_+/IL-1*β*+ cells was larger than the A*β*_1*–*42_−/IL-1*β*+ cell population (Figure 7(a)).

Pretreatment with Protollin increased the number of IL-1*β*+ cells displaying A*β*_1*–*42_ uptake (Figure 7(a)). Significant differences were observed between A*β*_1*–*42_+/IL-1*β*+ cells and A*β*_1*–*42_−/IL-1*β*+ cells treated with Protollin at 24 hours in concentrations of 0.001 *μ*g/mL (*P* < .05), 0.1 *μ*g/mL (*P* < .05) and 1 *μ*g/mL (*P* < .05), at 48 hours in concentrations of 0.1 *μ*g/mL (*P* < .05) and 1 *μ*g/mL (*P* < .05), and at 72 hours in a concentration of 0.1 *μ*g/mL (*P* < .05). 

### 3.9. IL-1RI Immunoreactive Cells

In untreated (vehicle) cultures the proportion of CHME3 microglial cells with immunoreactivity for IL-1RI (IL-1RI+) had a median value of 12.5% at 24 hours. Incubation with 1 *μ*g/mL A*β*_1*–*42_, with or without pretreatment with Protollin, did not significantly affect the number of IL-1RI immunoreactive cells at any time point (Figure 7(b)). 

When incubated with A*β*_1*–*42_ alone, the population of A*β*_1*–*42_+/IL-1RI+ cells was significantly larger than the A*β*_1*–*42_−/IL-1RI+ population at 24, 48, and 72 hours (*P* < .05, Figure 7(b)). When pretreated with Protollin, there was a significantly larger proportion of A*β*_1*–*42_+ cells that were immunoreactive to IL-1RI (A*β*_1*–*42_+/IL-1RI+) at 24 hours in concentrations of 0.001 *μ*g/mL (*P* < .05), 0.1 *μ*g/mL (*P* < .05) and 1 *μ*g/mL (*P* < .05), at 48 hours in concentrations of 0.1 *μ*g/mL (*P* < .05) and 1 *μ*g/mL (*P* < .05) and at 72 hours in a concentration of 0.1 *μ*g/mL (*P* < .05).

### 3.10. iNOS Immunoreactive Cells

In untreated (vehicle) cultures the median proportion of CHME3 microglial cells with immunoreactivity to iNOS (iNOS+) had a median of 16% at 24 hours under basal (vehicle) conditions. None of the treatments affected the total number of cells immunoreactive for iNOS (Figure 7(c)).

When incubated with A*β*_1*–*42_ alone, the population of A*β*_1*–*42_+/iNOS+ cells was significantly larger than the population of A*β*_1*–*42_−/iNOS+ cells at all time points (*P* = .0173 at 24 hours, *P* = .0172 at 48 hours, *P* = .0117 at 72 hours, and *P* = .05 at 96 hours, Figure 7(c)). Also when cells were pretreated with Protollin, the A*β*_1*–*42_+/iNOS+ population was significantly larger than the A*β*_1*–*42_−/iNOS+ population, at all concentrations tested (0.001 *μ*g/mL: *P* = .0117 at 24 hours, *P* = .0357 at 48 hours and *P* = .025 at 72 hours; 0.01 *μ*g/mL : *P* = .0117 at 24 hours and *P* = .0117 at 48 hours; 0.1 *μ*g/mL : *P* = .0117 at 24 hours, *P* = .05 at 48 hours and *P* = .0251 at 72 hours: and *P* = .025 at 72 hours: 0.1 *μ*g/mL : *P* = .0117 at 24 hours, Figure 7(c)).

### 3.11. Correlation between Secretion of BDNF and Uptake of A*β*_1–42_

**Figure 8:**
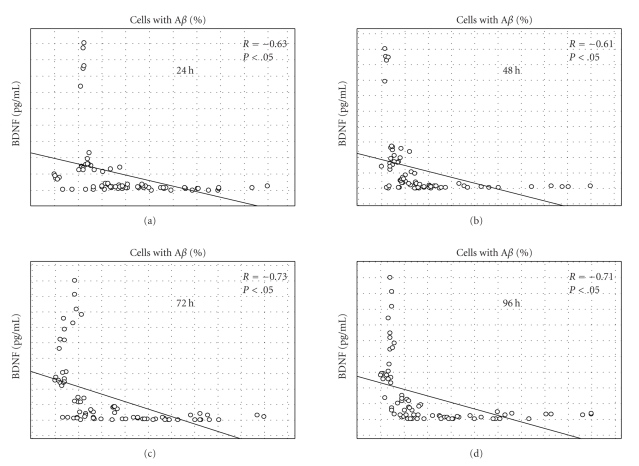
Correlation between the secretion of BDNF and the proportion of A*β*_1*–*42_+ cells. The secretion of BDNF (pg/mL) and the proportion of A*β*_1*–*42_+ cells in the Protollin series of experiments were analysed at each time point using Spearman's Rank Order Correlations test. A significant negative correlation was found at all time points. A statistical significant correlation is indicated by *(*P* < .05).

We found a significant negative correlation (*P* < .05) between the levels of BDNF in culture supernatant and the proportion of A*β*_1*–*42_+ cells at all time points in the experiments (Figure 8). The data were analysed by correlating the BDNF-levels with the proportion of A*β*_1*–*42_+ cells in all the treatments at one time point, and in one treatment at one time point, using Spearman's Rank Order Correlation test. There was a significant negative correlation in all analyses performed, except at 48 hours, when the treatment with 0.001 mg/mL Protollin was void of a significant correlation.

### 3.12. Cell Death and Viability

The effects of A*β*_1*–*42_ and the immunomodulatory substances on cell death and viability were analysed by the LDH and MTT assays, respectively. Neither A*β*_1*–*42_ nor Protollin, nor their combination, produced any significant effects on cell viability (data not shown).

**Figure 9:**
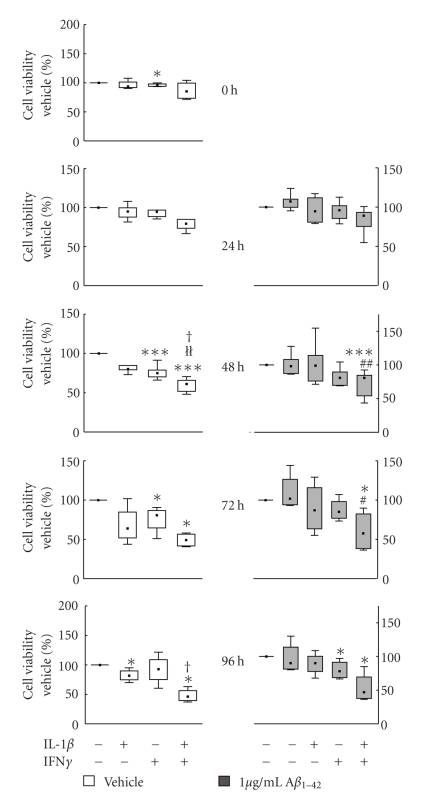
Effects of interleukin-1*β* (IL-1*β*) and interferon-*γ* (IFN*γ*) on cell viability in human CHME3 microglial cells treated with A*β*_1*–*42_ (grey boxes) or vehicle (white boxes). The cells were preincubated with 50 ng/mL IL-1*β* and 50 ng/mL IFN*γ* for 24 hours prior to addition of A*β*_1*–*42_ (1 *μ*g/mL). At 24, 48, 72, and 96 hours after addition of A*β*_1*–*42_ the viability of the cultures was assessed with the MTT assay. The data are expressed as % of control (vehicle) set at 100% and shown as median ± percentiles (25%–75% and 10%–90%), *n* = 4 (24, 48 h), *n* = 6 (72, 96 h). Statistical difference from vehicle is indicated by *(*P* < .05) and ***(*P* < .005), and statistical difference from A*β*_1*–*42_ is indicated by ^#^(*P* < .05) and ^##^(*P* < .01). 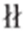
(*P* < .01) indicates statistical difference between IFN*γ* and IL-1*β* + IFN*γ*, and ^†^(*P* < .05) indicate differences between IL-1*β* and IL-1*β* + IFN*γ*.

The incubation with IFN*γ* or IL-1*β* + IFN*γ* decreased the signal from the MTT assay significantly to almost 50% of vehicle starting at 48 hours (*P* < .05), and at 72 (*P* < .005) and 96 hours (*P* < .005) (Figure 9). This effect was seen both when the microglia were stimulated with the cytokines alone and when they were added before A*β*_1*–*42_.

There were no significant differences in the LDH-activity in the medium from any of the treatments (not shown). Inspection with microscope, however, showed signs of cell death in treatments associated with a significant decrease in MTT signal.

## 4. Discussion

In this study, a human microglial cell line was used to model phagocytosis of A*β* and to evaluate the effects of different substances. The ability to analyse the effects of different substances with a human microglial cell line, which can be cultured in significant quantities, and the cellular reactions to pathological factors, such as A*β*, is a valuable tool in studies of human CNS-pathologies. The CHME3 cell line was established by Professor Tardieu (see [37]). This cell line responds to stimulation with LPS by increased secretion of IL-6 [38], a cytokine also secreted under basal conditions. We have found that CHME3 microglial cells also secrete low, but detectable, levels of IL-1*β* and TNF*α* as well as other cytokines (unpublished observations).

Phagocytosis of A*β*_1*–*42_ was established using fluorophore-labelled A*β*_1*–*42_. The uptake was first detectable at 4 hours after the addition as seen by microscopical analysis of living cells. Phagocytosis of A*β*_1*–*42_ was differentiated from unspecific adherence to cell membranes by confocal microscopy which confirmed the lysosomal location of A*β*_1*–*42_. In terms of inflammatory response, the incubation with A*β*_1*–*42_ at the concentration used (1 *μ*g/mL) did not affect the basal secretion of IL-6, in accordance with studies on primary human embryonic microglia in which A*β*_1*–*42_ failed to increase transcription of the IL-6 gene and A*β*_25*–*35_ had no effect on IL-6 secretion [39]. Similarly, the secretion of IL-6 from rat microglial cells was not altered upon incubation with 75 *μ*M A*β*_1*–*42_, a concentration 150-fold the concentration used in this study [18]. In contrast, primary mouse microglia have been shown to respond with markedly increased IL-6 secretion upon stimulation with A*β*_1*–*42_ in a concentration similar to that in the present study [40], indicating a species difference. In our previous studies on the CHME3 microglia a marked increase in the secreted levels of IL-6 could be seen upon incubation with A*β*_1*–*40_ [38], suggesting a difference in the immune-activating properties of the two forms of A*β*. 

We show here that the human microglial cell line produces and secretes BDNF, in agreement with studies on human *post mortem* tissue [41]. Studies in different injury models in animals have shown the induction of BDNF production in microglia [42], supporting a view of BDNF as a glial response to neuronal injury serving to help neurons to recover. In this context it is remarkable that the incubation with A*β*_1*–*42_ reduced the secretion of BDNF, which to our knowledge is the first time that A*β*_1*–*42_ has been shown to exert this effect on any cell type. This adds to the negative effects of A*β*_1*–*42_ and indicates a further reason for limiting the amyloidosis in AD. Evidence for detrimental interference by A*β*_1*–*42_ on BDNF signalling in neurons has been provided previously [43]. A reduced secretion and an impaired signalling of BDNF may contribute to the cell death and impaired neuronal function in AD. In fact, decreased levels of BDNF have been observed in the CSF of AD patients, and at later stages of the disease this reduction correlated with the severity of impairment [44].

Interestingly, a marked decrease in basal BDNF secretion was observed also upon incubation of the microglial cells with IFN*γ*, an effect that was also observed upon coincubation with IL-1*β* *.* In contrast, studies on rodent microglia showed an increase in the levels of BDNF in association with induction of inflammation [42], again suggesting species differences in microglial responses. Furthermore, studies on rat astrocytes indicated a stimulating effect on BDNF production by TNF*α* mediated by NF*κ*B [45]. In the present study IL-1*β*, which is also known to activate NF*κ*B [46], caused a mild inhibitory effect on BDNF secretion at 96 hours.

To analyse the possibility to stimulate the phagocytosis of A*β*_1*–*42_, we analysed the effects of different immunomodulatory substances. Pretreatment of the human microglial cells with IFN*γ*, alone or together with IL-1*β*, resulted in a significant increase in the proportion of cells with an uptake of A*β*_1*–*42_. This was accompanied by a pronounced reduction in BDNF secretion, as well as decreased cell viability. The results indicate that proinflammatory factors may be able to stimulate A*β*_1*–*42_ phagocytosis. However, the stimulation by IFN*γ* was seen in the later time points and it may be speculated that the acute inflammation at the start of the experiment had expired at this stage with presumably only low levels of the added cytokines still present in the medium. The effects observed may thus be due to factors induced by and secondary to IFN*γ*. The effect of long-term, chronic exposure to inflammatory stimulation may be different. The influence of inflammation on A*β*_1*–*42_ phagocytosis and clearance is a complex matter, where factors such as age and species may be pivotal. Even more complexity is added by the effects of A*β*_1*–*42_ itself on inflammation, including the findings that the 40 and 42 amino acid peptides, and the aggregational form of A*β*_1*–*42_ (monomers, oligomers, protofibrils, etc.), induce different inflammatory responses as seen in studies on rat microglia [18]. Furthermore, the shorter and longer species of A*β* have not been characterized with regard to their influence on glia. In the present study we prepared A*β*_1*–*42_ by dissolving the lyophilized peptide in pure DMSO, and therefore it is reasonable to assume that soluble monomers or oligomers dominate at the start of the experiments. Interestingly, we could not detect any significant difference in the proportion of A*β*_1*–*42_+ cells with time (data not shown). Factors influencing this proportion may be the rate of phagocytosis, degradation of the peptide, or changes in cell number. Our data show an ongoing cell proliferation continuing to the end of the experiment. This fact, taken together with a stable A*β*_1*–*42_+ cell proportion with time, suggests that phagocytosis is continuous. An exception was seen upon treatment with IFN*γ*, in which the A*β*_1*–*42_+ proportion increased significantly simultaneous with a reduction in cell viability. 

Protollin, a proteosome-based adjuvant with immunomodulatory activities, was found to modestly increase A*β*_1*–*42 _ uptake by the human CHME3 microglia at 96 hours after addition of A*β*_1*–*42_, but not at the earlier time points and only at the lowest concentration tested (0.001 *μ*g/mL). This potentially stimulatory effect of Protollin on A*β*_1*–*42 _ uptake is concordant with results from in vivo studies on Protollin in an AD mouse model [34], showing clearance of amyloid plaques and improved cognitive performance upon intranasal administration of Protollin. The limited effect of Protollin in the present in vitro experiments as compared to the in vivo studies may have several explanations. In the mouse in vivo model, the stimulatory effect of Protollin on A*β*-uptake appears to be mediated via activation of monocytes in the periphery that migrate to the brain and phagocytose A*β*, rather than direct activation of microglial cells resident in the brain [34]. Also, there was no evidence from the in vivo studies that Protollin translocates to the brain following nasal administration [34]. The modest stimulatory effect of Protollin on A*β*_1*–*42 _ uptake by microglial cells in the present study may reflect the absence of accessory cells in the in vitro cultures, that is, cells that Protollin directly activates in the periphery in vivo. Alternatively, considering that the effect of Protollin did not become apparent until the later part of the experiment, it may also be speculated that activation of microglial cell precursors by Protollin in the periphery results in the secretion of factors in a para/autocrine fashion, that with time build up to concentrations that stimulate phagocytosis. Species differences should also be considered. The response repertoire of glial cells appears to be different in human as compared with murine cells [47, 48]. 

Interestingly, the pretreatment with Protollin appeared to ameliorate the A*β*_1*–*42_-induced decrease in BDNF secretion. This highlights a therapeutic target: stimulation of glial cells for the production of beneficial and neuroprotective molecules. As indicated above, beneficial effects of Protollin in the context of immunotherapy may be elicited through stimulation of peripheral monocytes [34], directly, or indirectly through interaction with other immunocompetent cells. The present data, for example, on BDNF, suggest that Protollin or similarly acting substances may also stimulate beneficial effects on glial cells within the brain.

To our knowledge, no studies on human cells have until now investigated the effects of TLR2 activation on BDNF secretion. A strong negative correlation between the A*β*_1*–*42_+ cell proportion and the secretion of BDNF was also observed. This result can be due to a negative effect on phagocytosis by BDNF or a decrease in BDNF secretion by cells performing phagocytosis. In a previous study BDNF was found to stimulate phagocytosis [49]. Although the experiments were performed on mouse peritoneal macrophages, the results support the latter explanation for the negative correlation between BDNF levels and phagocytosis. 

Analysis with flow-cytometry showed that cells displaying phagocytosis of A*β*_1*–*42_ had a significantly higher degree of expression of IL-1RI and iNOS, indicating that phagocytosis of A*β*_1*–*42_ was associated with an inflammatory phenotype. Similarly, in a mouse AD-model, the expression of CD11b, a microglial activation marker that has been used as an indicator of harmful inflammation in several studies, was associated with removal of A*β* [34].

Protollin appeared to decrease the proportion of iNOS+ cells displaying phagocytosis of A*β*_1*–*42_, although the total number of iNOS+ cells remained unchanged. This proportion was significantly higher at all time points when incubated with A*β*_1*–*42_ alone. At 96 hours, the pretreatment with Protollin abolished this difference. A reduction in the levels of iNOS is beneficial due to the contribution of this enzyme to oxidative stress, supporting beneficial effects of Protollin.

The decrease in cell viability accompanying the induction of A*β* phagocytosis by IFN*γ* and IL-1*β* suggests microglial cell death. This was confirmed by microscopical inspection of the cultures. In spite of this, there was no detectable increase in the LDH-activity. However, a decrease in cell number as indicated by the MTT-assay could mask an increase in cell death as measured by the LDH-assay since fewer cells are available to release LDH into the medium. IFN*γ* has been shown to induce cell death in murine microglia, concomittant with an upregulation of the expression of Fas and FasL [50]. In addition, IL-1*β* and IFN*γ* are known to be involved in the expression and activation of iNOS [51, 52], which in turn may lead to oxidative stress.

In conclusion, we provide a model suitable for testing candidates for stimulating the phagocytosis of A*β*_1*–*42_ by human microglial cells. The capacity to withstand serum-withdrawal for long periods of time and the high rate of proliferation makes the human CHME3 cell line suitable for this type of studies. Expanding primary cultures of microglia for large experimental series is not trivial. The data presented indicate differences between human microglial cells and murine glia, as described in other studies. In the present study, we show that the immunomodulatory substance Protollin affects the proportion of cells phagocytosing A*β* and their expression of inflammatory markers. Pretreatment with IFN*γ* had a robust stimulatory effect on phagocytosis of A*β*_1*–*42_ at later time points, suggesting influence of secondary factors induced by this cytokine. In addition, we present data suggesting a neuropathological role for IFN*γ* by decreasing BDNF levels. Importantly, we show a strong negative effect of A*β*_1*–*42_ on BDNF secretion. If phagocytic cells indeed have a decreased secretion of BDNF, as indicated by the negative correlation between phagocytosis and BDNF, this suggests an interesting parameter for future evaluation of potential drugs. The results present a scenario in which inflammation increases phagocytosis of A*β*_1*–*42_, induces microglial cell death, and reduces secretion of BDNF. The reduction in BDNF is unwanted, since it deprives the brain of an important neuroprotective and plasticity-promoting factor. Considering the presence of inflammation in AD, it may be suggested that the combined effect of A*β*, IL-1*β*, and IFN*γ* on the secretion of BDNF from microglia may contribute to the neuronal pathology in AD. We hope that this and future studies can help develop directed and controlled activation of differential pro- and anti-inflammatory responses for therapeutic use.
